# 

**Published:** 1940

**Authors:** 


### CONTENTS.

| | Page |
|---|---|
| **Electro-Encephalography: An Aid to Diagnosis.** By W. Grey Walter, M.A. | 1 |
| **Malum Immedicabile Cancer.** By E. Watson-Williams, M.C., Ch.M. | 9 |
| **University of Bristol Dental Hospital and School** | 41 |
| **Reviews of Books** | 46 |
| **Editorial Notes** | 54 |
| **Obituaries:** Julia Woolnough Gross | 58 |
| George Henry Temple, M.B., C.M. | 59 |
| Elliott Thornton Glenny, M.B., B.S.Lond., M.R.C.S., L.R.C.P. | 61 |
| **Meetings of Societies** | 62 |
| **The Medical Library of the University of Bristol** | 62 |
| **Publications Received** | 66 |
| **Local Medical Notes** | 67 |

